# On the electrostatic component of protein-protein binding free energy

**DOI:** 10.1186/1757-5036-1-2

**Published:** 2008-11-05

**Authors:** Kemper Talley, Carmen Ng, Michael Shoppell, Petras Kundrotas, Emil Alexov

**Affiliations:** 1 Computational Biophysics and Bioinformatics, Department of Physics, Clemson University, Clemson, SC 29634, USA; 2 James Byrnes High School, Duncan, SC 29334, USA; 3 South Carolina Governor School for Science and Mathematics, Hartsville, SC 29550, USA; 4 Center for Bioinformatics, The University of Kansas, Lawrence, KS 66047, USA

## Abstract

Calculations of electrostatic properties of protein-protein complexes are usually done within framework of a model with a certain set of parameters. In this paper we present a comprehensive statistical analysis of the sensitivity of the electrostatic component of binding free energy (ΔΔG_el_) with respect with different force fields (Charmm, Amber, and OPLS), different values of the internal dielectric constant, and different presentations of molecular surface (different values of the probe radius). The study was done using the largest so far set of entries comprising 260 hetero and 2148 homo protein-protein complexes extracted from a previously developed database of protein complexes (*ProtCom). *To test the sensitivity of the energy calculations with respect to the structural details, all structures were energy minimized with corresponding force field, and the energies were recalculated. The results indicate that the absolute value of the electrostatic component of the binding free energy (ΔΔG_el_) is very sensitive to the force field parameters, the minimization procedure, the values of the internal dielectric constant, and the probe radius. Nevertheless our results indicate that certain trends in ΔΔG_el _behavior are much less sensitive to the calculation parameters. For instance, the fraction of the homo-complexes, for which the electrostatics was found to oppose binding, is 80% regardless of the force fields and parameters used. For the hetero-complexes, however, the percentage of the cases for which electrostatics opposed binding varied from 43% to 85%, depending on the protocol and parameters employed. A significant correlation was found between the effects caused by raising the internal dielectric constant and decreasing the probe radius. Correlations were also found among the results obtained with different force fields. However, despite of the correlations found, the absolute ΔΔG_el _calculated with different force field parameters could differ more than tens of kcal/mol in some cases. Set of rules of obtaining confident predictions of absolute ΔΔG_el _and ΔΔG_el _sign are provided in the conclusion section.

PACS codes: 87.15.A-, *87.15. km*

## Introduction

Proteins are essential components of the living cell[1]. They function by interacting with other biological macromolecules, including other proteins. Therefore, understanding protein-protein interactions is a very important step in learning about cell function, which in turn requires understanding the forces and effects that influence these interactions [2-10]. Protein-protein binding is a complex process mostly driven by the hydrophobic effect and van der Waals interactions, with significant contribution from entropy and electrostatics. While these energies can not be easy experimentally separated, the electrostatic energy is the energy mostly affected by pH and salt concentration. The interest of modeling pH and salt dependent phenomena inspired many researchers to model the electrostatic component of the binding free energy alone [11]. Nowadays, the 3D structures of a significant number of protein-protein complexes are available, which allows researchers to model the contribution of electrostatic energy to the binding [12-19]. Experimental [20-23] and computational[24] mutagenesis studies have found that most of the ionizable residues at protein-protein interfaces contribute significantly to the binding free energy (i.e., replacement of those residues with the alanine residues critically affects the protein binding affinity). In many cases, the formation of a complex creates favorable pair-wise electrostatic interactions across the interface, as was demonstrated in a series of work on the barnase-barstar complex[17,25-28] and on other complexes[19,29-33]. Authors of several studies[7,34,35] have concluded that electrostatic interactions have a dominant contribution into total binding free energy for the complexes with small interfaces. The contribution of electrostatic energy to the binding affinity of the particular, Rap/Raf, complex was the subject of a series of investigations[36,37]. Despite the fact that all of the abovementioned studies agreed that there are many specific pair-wise electrostatic interactions across the interface, the conclusions about the role of electrostatics on binding affinity remain controversial. It was found that, in some cases, electrostatic energy favors binding, while in other cases, it opposes it. Since the electrostatic component of the binding free energy is the difference between two large and similar numbers (energy of pair-wise interactions and the desolvation penalty), the calculations outcome inevitably depends on parameters of simulations such as the force field parameters, the choice of the internal dielectric constant of proteins, and the molecular surface representation. In addition, structural refinement also affects the outcome of electrostatic calculations.

The internal dielectric constant is an important characteristic of protein's polarizability [38-41]. However, it was repeatedly shown in the literature that the dielectric constant is neither a constant nor is there an optimal value that works for all cases. Molecular dynamic simulations with implicit solvent models use a dielectric constant of either 1.0 or 2.0[42], the molecular mechanics Poisson-Boltzmann (MMPB) method usually employs a dielectric constant of 2.0[43,44], and pKa's calculations are usually carried out with a dielectric constant larger or equal to 4.0[45,46]. In our study the dielectric constant will be considered a parameter, and we will vary its value to reveal the sensitivity of the calculations of the electrostatic component of binding. The other parameter that will be varied is the probe radius. As pointed out by Zhou and co-workers[17,47,48], the electrostatic component of the binding free energy is very sensitive to the method used to build the molecular surface. Recently, the effect of different presentations of the molecular surface was tested on a set of 64 mutants[47], which demonstrated that calculations done with van der Waals molecular surface give better agreement with experimental data delivered from a double mutant cycle. The charge distribution and atomic radii greatly affect numerical calculations as well[49]. Currently, there are many force fields, each having different partial charge distributions and sets of atomic radii. Three of the most popular force fields, Charmm, Amber, and OPLS will be used in this study to reveal the sensitivity of the calculations with respect to the force field parameters. Previous work has shown that optimal hydrogen bonding is crucial for correct predictions of pKa's of ionizable groups[50,51]. However, different force fields generate different hydrogen networks[52]. In addition, the calculated energies strongly depend on structural details, and many studies have been done on refined structures[53], i.e., on structures that were subjected to energy minimization. Therefore, it is desirable to know what will be the effect of minimization on the calculations of the electrostatic component of binding, if minimization is done with different force fields.

In this study we test the sensitivity of the electrostatic component of the binding free energy with respect to different force field parameters, structural relaxation, values of the internal dielectric constant, and probe radii. The study will be done on a large set of protein-protein complexes comprised of 260 hetero- and 2148 homo-complexes. Such a large dataset has never been investigated before, especially with energy minimized structures.

## Methods

### Protein-protein complexes used in the study and their parameters

Protein-protein hetero-complexes subjected to the study were extracted from the *ProtCom*[54] database (as of November 2006) , which contains 1771 entries at 95% sequence identity level. To avoid the bias toward overrepresented complexes, the entries were purged with *CD-hit*[55] at 40% sequence identity level for all components of the hetero-complexes, including monomers that belong to the same protein-protein complex. This resulted in 299 structures out of which 39 structures were excluded from further consideration due to large defects in the PDB files (like large segments of missing polypeptide chains, for example), which constitute untreatable obstacles for the correct protonation of the structures. The structures of homo-complexes were taken from 40% sequence identity of the *ProtCom *database. Some structures were excluded because of large structural defects, which reduced our homo-complex set to 2617 structures at 40% sequence identity level.

This resulted in an initial set of 260 hetero- and 2617 homo-protein complexes, which after removing structures for which some of the computational procedures could not produce the desired accuracy was reduced to 260 hetero- and 2148 homo-protein complexes. The interfacial area was calculated with the *surfv *program[56] by subtracting the accessible surface area of the complex from the accessible surface area of the free monomers. The net charge of the complex and the free monomers were calculated assuming that all titratable amino acids were in their charged state.

### Hydrogen placement and energy minimization

Some of the structures in our initial data set had structural defects, and thus all of the structures were subjected to the *profix *program from the Jackal package developed in Honig's lab (: Jackal) in order to add missing atoms and/or sequence fragments. All of the complexes that had a missing segment chain longer than fifteen amino acids were removed from the set because building such long sequence stretches could introduce significant structural errors. The rest of the structures were subjected to the TINKER[57] software to add missing hydrogens using the package's *pdbxyz.x *and the *xyzpdb.z *modules with three different force field parameters: Charmm27[58], AMBER[59], and OPLS[60]. We will refer to this set of structures as the non-minimized set.

A common approach in computing biophysical quantities using the 3D structures of biological macromolecules is to refine these structures by performing energy minimization with a particular force field. Following this strategy, we created another set of protonated structures (hereafter referred to as the minimized set) for all of the PDB files described above by running the *minimize.x *module of the TINKER software between running the *pdbxyz.x *and the *xyzpdb.z *modules. The *minimize.x *module performs energy minimization using the Limited Memory BFGS Quasi-Newton Optimization algorithm[57]. The implicit solvent was modeled using the Still Generalized Born model[61], and the internal dielectric constant was set to 1.0 to be consistent with the corresponding force field parameters[62] used in the calculations. To test the sensitivity of the results, the minimizations were done with Charmm27[58], AMBER[59], and OPLS[60] parameters. A weak convergence criteria (RMS gradient per atom = 0.1) was applied to make computation tractable. Even in such a case, minimizing 2408 protein-protein complexes, some of which were larger than 50,000 atoms, was quite a challenge from a computational point of view (monomers were not minimized separately and their structures were taken from minimized complexes). Therefore, for these calculations we utilized a high throughput distributed computing resource, *CONDOR*, originally developed at the University of Wisconsin-Madison , which is available at Clemson University with more than 2,000 single CPUs of computational power.

### Calculation of the electrostatic component of the binding free energy

The electrostatic component of the binding free energy (ΔΔG_*el*_) was calculated as the difference of the electrostatic free energies of the complex and of the free molecules[63]:

(1) ΔΔ*G*_*el *_= Δ*G*_*el*_(*A*:*B*) - Δ*G*_*el *_(*A*) -Δ*G*_*el*_(*B*)

where Δ*G*_*el*_(*X*)is the electrostatic component of the folding energy of the complex, monomer A and monomer B, respectively (X = AB, A or B). The 3D structures of the monomers were taken from the corresponding complex, i.e. no conformational changes associated with the binding were modeled. Since the 3D structures of the bound and unbound monomers are the same, their internal mechanical energies are the same as well and do not affect the calculations. These energies were calculated as a sum of Coulombic interactions and reaction field energies, as discussed in detail in Ref.[64]. The Coulombic energy was calculated in homogeneous media with a dielectric constant equal to the internal dielectric constant of the protein. The reaction field energy was calculated as the energy of the interaction of permanent charges with induced charges at the surface of the corresponding entity – protein complex or a monomer. These energy terms were calculated with the Delphi[64,65] program using a grid size of 129. The calculations were performed as zero ionic strength. The external dielectric constant was kept at 80. The convergence criteria was rmsc = 0.0001 kT/e (see the Delphi manual for details at : Delphi).

The calculations were performed assuming that all of the Arg, Asp, Glu, and Lys residues were ionized in both free and bound states, while the His residues were considered neutral. In order to reduce the complexity of the problem, all ionizable residues were kept in their default charge state. Thus, ionization changes induced by complex formation (suggested either by experimental data[66,67] or by theoretical simulations [68-71]) were not considered.

### Parameters that will be varied

#### Force field parameters

Three force field parameters were used in this study: Charmm27[58], AMBER[59], and OPLS[60]. These force fields are all atom force fields but were parameterized differently.

#### Internal dielectric constant

What the value of the internal dielectric constant (ε(in)) should be for a given protein is the subject of much debate in the scientific community[41]. In this study we adopt a pragmatic approach and test the sensitivity of the results at ε(in) = 1, 2, 4, 8, 20.

#### Probe radius

The molecular surface in FDPB algorithms is determined by rolling a water probe with a particular radius to find the molecular surface according to Richard's algorithm[72]. A typical probe radius is 1.4A. However, Zhou and co-workers[73] have introduced the concept of a zero probe radius, and here we will explore the effect of different probe radii on the electrostatic component of the binding free energy. The probe radius will vary taking values of R = 0.0, 0.5, 1.0, and 1.4A.

## Results

Two types of complexes, hetero- and homo-complexes are the subjects of our study. We will explore how similar and how different are they with respect to their macroscopic characteristics. Since the physical process of binding is governed by the same forces, it may be expected that their physico-chemical properties should be quite similar. Table 1 summarizes the global and interfacial properties of the complexes used in our study. It can be seen that the interfacial areas of the hetero- and homo-complexes are quite similar in terms of their maximal and minimal sizes. The mean of the distribution of the interfacial area of the hetero-complexes is about 200A larger than that of the homo-complexes. The same is observed for the number of interfacial residues; the mean of the distribution is slightly larger for the hetero-complexes. Minimum and maximum of both the net and interfacial charges are quite similar for the hetero- and homo-complexes. The main difference is observed in terms of electrostatic pairing. By the virtue that homo-complexes are made of identical monomers, all homo-complexes are made of monomers carrying the same polarity charge. In contrast, 40% of the hetero-complexes are made of monomers having an opposite net charge. The same analysis done for the interfacial charges confirms the electrostatic difference between the hetero- and homo-complexes. Only 8% of the homo-complexes form interfaces carrying opposite charges, while the charges of the interfaces are opposite in 58% of the hetero-complexes. The global and interfacial differences of the charges among the hetero- and homo-complexes suggest that electrostatics could play different roles in hetero- and homo-complexes association. Because of that, the results for the hetero- and homo-complexes will be reported separately.

**Table 1:** Macroscopic parameters of hetero- and homo-complexes

	Hetero-complexes	Homo-complexes
Max interfacial area (A**2)	8223.6	9595.2
Min interfacial area (A**2)	275.7	252.5
Mean interfacial area (A**2)	1440.2	1260.7
Max interfacial residues	408	461
Min interfacial residues	15	12
Mean interfacial residues	77	73
Max/min net charge	+15/-48	+23/-53
Max/min interfacial charge	+13/-14	+10/-16
Percentage having opposite net charge of the monomers	40%	0%
Percentage having opposite interfacial change of the monomers (in parentheses are the cases for which at least one of the interfaces had zero net charge)	58% (23% zero)	8% (33% zero)

The files are taken from 40% sequence identity subset of the ProtCom database.

### Effects of the internal dielectric constant

**Figure 1:**
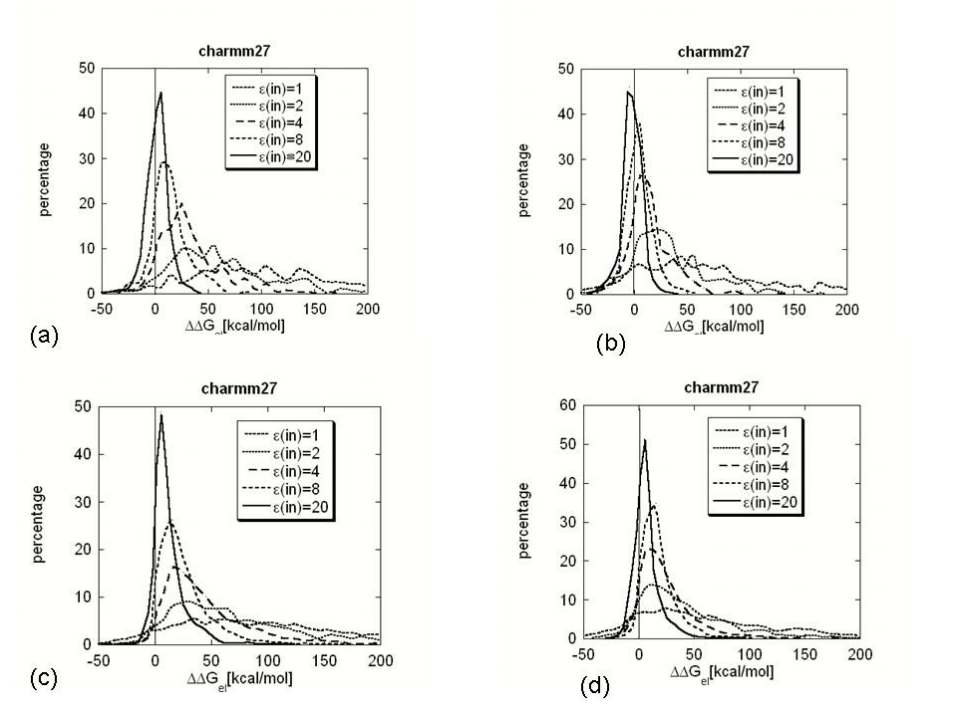
Distribution of the electrostatic component of the binding energy (ΔΔG_el_) calculated with dielectric constant ε(in) = 2.0, probe radius of 1.4A and Charmm27 force field. Percentage is the count of ΔΔG_el _normalized in respect with the total number of complexes. The results are presented in case of: (a) non-minimized hetero-complexes. (b) minimized hetero-complexes. (c) non-minimized homo-complexes. (d)minimized homo-complexes.

In this study, the value of the dielectric constant was varied from one, which is the value usually used in molecular dynamics simulations with explicit water model, to twenty, which was introduced by Gilson and co-workers[46] as the best value for rigid-body pKa calculations. Further discussion of the role and optimal value of the internal dielectric constant is provided in series papers of Warshel and co-workers [41,74-76]. It is well known that electrostatic energy terms, even in case of irregularly shaped objects, are roughly inversely proportional to the value of the dielectric constant. Since the electrostatic component of the binding free energy is made of two major components, Coulombic and de-solvation energies, their difference should also be roughly inversely proportional to the value of the dielectric constant. The distribution of the electrostatic component of the binding free energy is shown in Fig. 1 for different values of the internal dielectric constant. These calculations were done with the Charmm27 force field parameters. It can be seen that using a low dielectric constant (ε(in) = 1, 2 and 4), the mean of the distribution for both the hetero- (Fig. 1a and 1b) and homo-complexes (Fig. 1c and 1d) is shifted toward positive ΔΔG_el_. For ε(in) = 1 and ε(in) = 2 in 94% of the non-minimized complex cases, the electrostatics is calculated to oppose binding. Minimization of the structures slightly lowers this percentage to 87%; however, in the vast majority of the cases, the calculated electrostatic component of the binding free energy still opposes binding. The corresponding numbers for homo-complexes are 91% and 85% for non-minimized and minimized complexes, respectively. These percentages are quite similar and show no significant difference between the electrostatic components of the binding free energy for the hetero- and homo-complexes. Further increasing the value of the internal dielectric constant makes ΔΔG_el _smaller and smaller, which results in a narrower distribution, but the majority of the energies remain positive. At the largest value used in this study (ε(in) = 20), in 62% of the non-minimized hetero-complex cases, the electrostatic component of the binding free energy was calculated to oppose binding. This percentage drops to 43% in case of minimized hetero-complexes. Quite different percentages were calculated in case of the homo-complexes. Raising the internal dielectric constant to twenty only minimally changed the percentage of the ΔΔG_el _calculated to favor binding; however, in at least 91% of the cases, ΔΔG_el _still opposes binding. Energy minimization had a significant effect, lowering the percentage of cases in which the electrostatic component of the binding free energy was calculated to be positive number to 77%. However, electrostatics was still calculated to oppose binding in the vast majority of the homo-complexes.

Why were the calculated distributions of ΔΔG_el _using the low internal dielectric constant quite similar for the hetero- and homo-complexes but significantly different using high values of the internal dielectric constant? A possible explanation is offered by the numbers reported in the last two rows of Table 1. From the results in Table 1, it can be concluded that in the vast majority of the cases, the homo-complexes form interfaces that carry the same polarity charge, while in the hetero-complex cases, more than 58% of the complexes have oppositely charged interfaces. Thus, in the vast majority of the cases, the Coulombic energy has a large positive magnitude for homo-complexes (interactions between the same polarity charges across interfaces), while this percentage is much smaller for hetero-complexes. This results in a larger amplitude of the calculated ΔΔG_el _for homo-complexes compared with the ΔΔG_el _calculated for the hetero-complexes. Increasing the internal dielectric constant combined with minimizing the energy of the structures could turn some of the small positive ΔΔG_el _calculated for the hetero-complexes into small negative ΔΔG_el_. However, ΔΔG_el _are large positive numbers in the homo-complexes set, and an increase of the internal dielectric constant combined with the refinement of the structures cannot dramatically change the percentage of the cases in which electrostatics was calculated to oppose binding.

The observation made for the hetero-complexes, that their structural refinement through the energy minimization of corresponding complexes results in a change of the sign of the calculated ΔΔG_el_, deserves attention. The electrostatics is one of the components of the total energy and the total energy is minimized in the minimization protocol. There is no reason to expect that minimizing the total energy should result in the minimization of the energy components. However, apparently the electrostatic component was actually optimized in the energy minimization protocol. Perhaps this indicates that if a given energy component favors a particular energy state, the minimization of the total energy will most likely result in the enhancement of this energy component as well.

**Figure 2:**
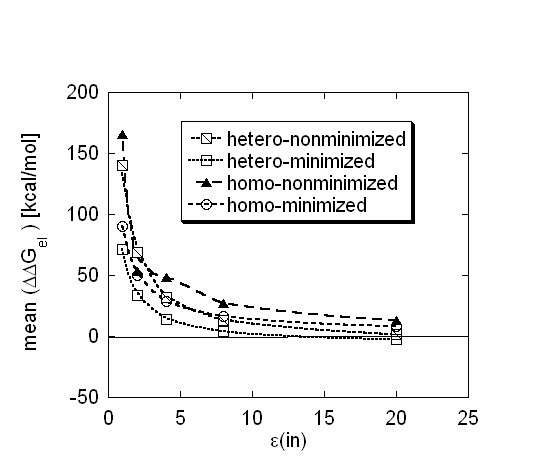
The mean of the ΔΔG_el _distributions calculated with at probe radius R = 1.4 A plotted as a function of the internal dielectric constant ε(in).

The results of this section are summarized in Fig. 2, where the means of the corresponding ΔΔG_el _distributions are plotted against the internal dielectric constant. The calculations were done with the probe radius R = 1.4A. It can be seen that variations of the internal dielectric constant within the range 1.0–8.0 cause dramatic changes in the mean of the energy distributions for all types of complexes. In contrast, an increasing the magnitude of the internal dielectric constant above 8.0 does not cause much change in the calculated ΔΔG_el_. Despite the changes of the distributions' mean magnitudes as the internal dielectric constant varies, the mean is always above zero for the homo-complexes and for the non-minimized hetero-complexes. The mean drops below zero only in minimized hetero-complex set when the internal dielectric constant is above 8.0.

### Effects of the probe radius

**Figure 3:**
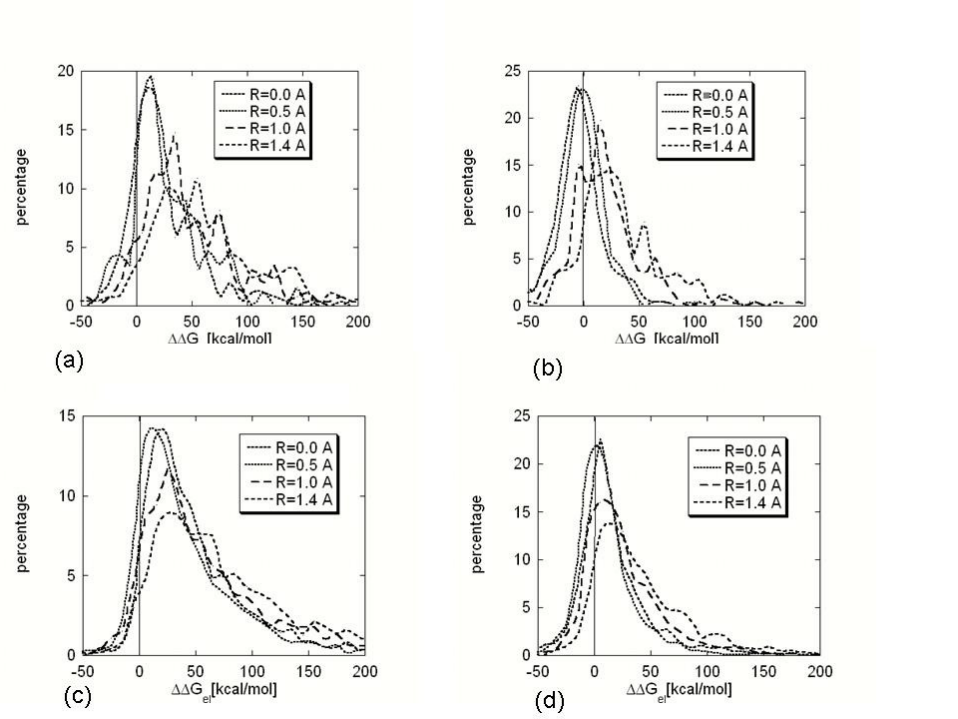
Distribution of the electrostatic component of the binding energy (ΔΔG_el_) calculated with dielectric constant ε(in) = 2.0, probe radius of 0.0A and Charmm27 force field. Percentage is the count of ΔΔG_el _normalized in respect with the total number of complexes. The results are presented in case of: (a) non-minimized hetero-complexes. (b) minimized hetero-complexes. (c) non-minimized homo-complexes. (d)minimized homo-complexes.

The probe radius in finite-difference algorithm plays an important role in determining both the molecular surface and the internal dielectric space of molecules[17,77]. A small probe radius results in a rough molecular surface and many artificial high dielectric cavities in the protein interior[17,77]. However, getting away from the physical interpretation of the probe radius value, we would like to look at it as a parameter and to investigate how it affects the electrostatic component of the binding free energy. The distribution of the electrostatic component of the binding free energy using an internal dielectric constant of 2.0 and different probe radii are shown in Fig. 3 for both hetero- and homo-complexes. It can be seen that decreasing the probe radius causes similar effects as does increasing the internal dielectric constant (compare Figs. 1 and 3). At probe radii of 1.4A and 1.0A, the distributions of both the hetero- and homo-complexes ΔΔG_el_, with and without minimized structures are offset towards positive energies, .i.e., in the vast majority (over 85%) of cases, the calculated energies oppose binding. As the probe radius magnitude decreases and reaches 0.5A and 0.0A, the distributions move to the left, towards negative ΔΔG_el_. However, the distributions of ΔΔG_el _for non-minimized hetero- and homo-complexes and for minimized homo-complexes remain mostly at positive energies. At a probe radius R = 0.0A, for 81% of the non-minimized hereto-complexes, the electrostatics was calculated to oppose binding. The percentage of the homo-complexes with positive ΔΔG_el _was 94% and 73%, for non-minimized and minimized complexes, respectively. The only exceptions are the distributions of ΔΔG_el _for minimized hetero-complexes at probe radii of 0.5A and 0.0A. At a probe radius of 0.0A, for 40% of the minimized hetero-complexes, the electrostatics was calculated to oppose binding. It is interesting to note that similar percentage were calculated for minimized hetero-complexes using a "standard" probe radius of 1.4A, but assigning a high internal dielectric constant of 20, resulted in 43% of the calculated energies being positive.

**Figure 4:**
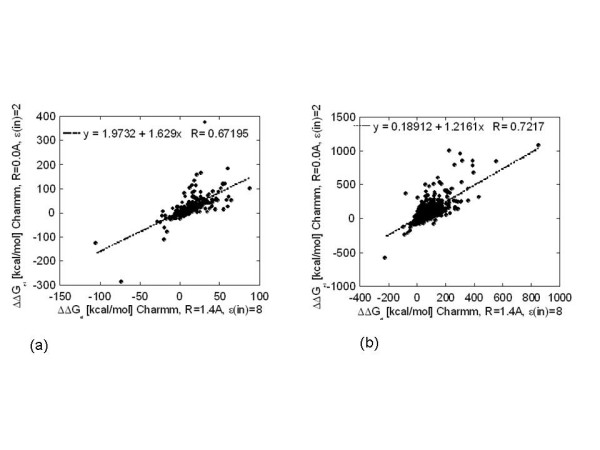
ΔΔG_el _calculated with probe radius 0.0A and ε(in) = 2.0 versus ΔΔG_el _calculated with "standard" probe radius 1.4A and different ε(in): (a) non-minimized hetero-complexes, ε(in) = 8.0. (b) non-minimized homo-complexes, ε(in) = 4.0.

The observed similarities of the effects caused by increasing the internal dielectric constant and decreasing the magnitude of the probe radius inspired us to investigate a possible correlation between the ΔΔG_el _calculated with a "standard" probe radius value but with a high internal dielectric constant and the ΔΔG_el _calculated with a small probe radius and a "standard" internal dielectric constant. Here by "standard" we mean values that are most frequently reported in the literature. To address such a possibility, the corresponding ΔΔG_el _for each type of dataset, hetero- and homo-complexes, non-minimized and minimized, were subjected to the following procedure: ΔΔG_el _calculated with probe radius 0.0A and 'standard" dielectric constant of 2.0 were plotted against ΔΔG_el _calculated with probe radius 1.4A and ε(in) = 1, 2, 4, 8 and 20. The plot resulting to largest correlation coefficient and a slope close to 1.0 was considered as the best fit. In case of non-minimized hetero-complexes (Fig. 4a) the best fit was obtained at ε(in) = 8.0 (Table 2, first row). In case of non-minimized homo-complexes, the best correlation between the calculations with probe radius of 0.0A and 1.4A was obtained at ε(in) = 4.0 (Fig. 4b and Table 2 third row). The same procedure was applied to minimized structures and the results in terms of the slope of the fitting line and the correlation coefficient are shown in Table 2 for hetero- and homo-complexes. Then the procedure was repeated to find the best fit between ΔΔG_el _calculated with slightly larger probe radius of 0.5A and 'standard" dielectric constant of 2.0 versus ΔΔG_el _calculated with probe radius 1.4A and ε(in) = 1, 2, 4, 8 and 20. The best fits in terms of correlation coefficient and slope are reported in Table 2, the last four rows. It can be seen that in some cases, the correlation coefficient reaches 0.87, and in other cases, the slope of the fitting line is close to 1.0. However, neither of the plots resulted in a fitting line slope of 1.0 with a simultaneous correlation coefficient of more than 0.90. Thus, despite the observed correlations, the effects caused by the variations in the magnitude of the internal dielectric constant and the probe radius are, strictly speaking, different and depend on particular cases being considered. Perhaps, more detailed sampling of different internal dielectric constant values could obtain better correlations.

**Table 2:** Slopes of the fitting lines and the corresponding correlation coefficients

Type of complex	Probe radius R = 1.4A and different ε(in)	ε(in) = 2 and different probe radius R	Slope of the fitting line	Correlation coefficient
Hetero, non-minimized	8.0	0.0	1.63	0.67
Hetero, minimized	8.0	0.0	1.31	0.59
Homo, non-minimized	4.0	0.0	1.22	0.72
Homo, minimized	4.0	0.0	1.23	0.85
Hetero, non-minimized	4.0	0.5	0.69	0.52
Hetero, minimized	4.0	0.5	0.92	0.79
Homo, non-minimized	4.0	0.5	1.44	0.87
Homo, minimized	4.0	0.5	1.34	0.85

The results are presented for the *best *correlations among ΔΔG_el _calculated with "standard" probe radius of 1.4A versus small probe radius of 0.0 and 0.5A. The *best *correlations are those resulting in the best correlation coefficient and slope close to 1.0. In obtaining the best fits, the internal dielectric constant e(in) was varied (ε(in) = 1,2,4,8,20) in the ΔΔG_el _calculations with "standard" probe radius of 1.4A.

**Figure 5:**
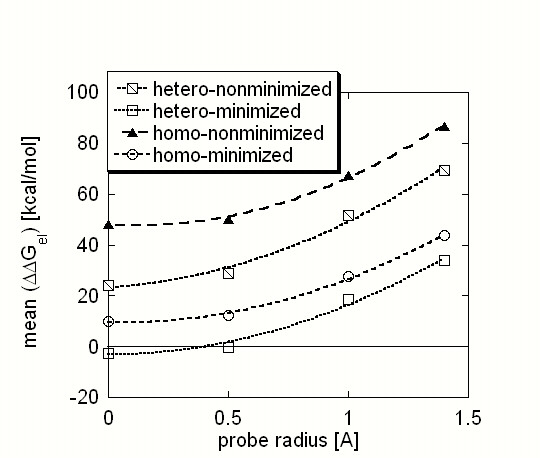
The mean of the ΔΔG_el _distributions calculated with internal dielectric constant ε(in) = 2.0 as a function of the probe radius.

The effect of the probe radius on the calculated ΔΔG_el _is summarized in Fig. 5, where the means of the corresponding ΔΔG_el _distributions are shown as a function of the probe radius. It can be seen that decreasing the magnitude of the probe radius results in a decrease of the mean of the ΔΔG_el _distributions. A similar trend was discussed in the previous paragraph regarding increasing the internal dielectric constant. The decrease of the mean is almost the same for all cases: for the hetero- and homo-complexes and for the non-minimized and minimized. However, the mean of the distribution of homo-complexes at R = 1.4A is much more positive than that of the hetero-complexes, and the decrease caused by lowering the radius is not enough to make it a negative number. In contrast, the change is enough to turn the sign of the mean of the minimized hetero-complexes and to make it negative.

### The effects of the force field

The above results were calculated using Charmm27 force filed parameters for both calculations of the electrostatic energies and of the minimization protocol. To test the sensitivity of the results with respect to the force fields, we repeated the calculations with Amber98 and OPLS force fields. This required energy minimization of both the hetero- and homo-complexes with Amber and OPLS force fields, respectively. The results are reported in the Supplementary Materials section, where the ΔΔG_el _calculated with Charmm27 are plotted against the corresponding ΔΔG_el _calculated with Amber98 and OPLS, respectively. The data points were fitted with straight lines and the scopes and correlation coefficients recorded. These slopes and correlation coefficients are provided in Tables 3, 4. In addition, we calculated the difference of the ΔΔG_el _calculated with different force fields as

**Table 3:** The parameters of the distributions of ΔΔΔG_el _calculated for hetero-complexes.

	Amber-Charmm	Amber-OPLS	Charmm-OPLS
Min	-36.6 (-33.8)	-41.5 (-75.9)	-33.7 (-183.5)
Max	54.3 (107.6)	75.4 (100.1)	56.7 (76.4)
Mean	5.8 (10.3)	13.0 (19.0)	7.3 (8.8)
Median	5.9 (8.6)	10.8 (16.3)	6.0 (7.3)
RMS	15.0 (17.0)	21.5 (27.7)	15.5 (23.6)
Variance	194 (183.4)	295 (412)	188 (481)
R	0.95 (0.94)	0.92 (0.90)	0.95 (0.89)
slope	0.96 (0.83)	0.89 (1.08)	0.91 (1.21)

The numbers without parentheses are for non-minimized and the numbers in parentheses are for minimized complexes. The last two rows report the correlation coefficient and the slope of the fitting line of the graphs provided in Additional file 1. The calculations were done with probe radius 1.4A and internal dielectric constant of 2.0.

**Table 4:** The parameters of the distributions of ΔΔΔG_el _calculated for homo-complexes.

	Amber-Charmm	Amber-OPLS	Charmm-OPLS
Min	-226.8 (-274.9)	-644.7 (-673.3)	-300.1 (-327.5)
Max	262.3 (166.4)	572.8 (438.8)	329.1 (269.4)
Mean	15.3 (-2.63)	-30.9 (-15.2)	2.6 (4.8)
Median	9.5 (-2.12)	-28.1 (-13.6)	0.7 (1.6)
RMS	32.6 (30.7)	70.4 (63.6)	27.7 (21.8)
Variance	829 (940)	4003 (3267)	768 (871)
R	0.92 (0.93)	0.78 (0.85)	0.96 (0.95)
slope	0.85 (0.81)	0.88 (0.89)	0.94 (0.93)

The numbers without parentheses are for non-minimized and the numbers in parentheses are for minimized complexes. The last two rows report the correlation coefficient and the slope of the fitting line of the graphs provided in Additional file 1. The calculations were done with probe radius 1.4A and internal dielectric constant of 2.0.

(2) ΔΔΔ*G*_*el *_(*X*: *Y*) = ΔΔ*G*_*el*_(*X*) - ΔΔ*G*_*el*_(*Y*)

where X, Y stand for either the Charmm27, Amber98 or OPLS force fields. These differences were calculated for all types of complexes, both non-minimized and minimized. The parameters of the resulting distributions are reported in Tables 3, 4. The corresponding graphs are shown in Additional file 1.

It can be seen that both the correlation coefficients and the slope of the fitting lines are close to 1.0, indicating that ΔΔG_el_s calculated with different force fields are quite similar. However, a closer look at Tables 3, 4 shows significant differences as well. The mean of ΔΔG_el _calculated with Charmm27 is 5.8 and is 10.3 kcal/mol less positive than that of the corresponding means calculated with Amber98 in the case of non-minimized and minimized hereto-complexes, respectively. The same offset is even more pronounced when comparing the results obtained with the Amber and OPLS force fields. In the hetero-complex cases, the means differ by 13.0 and 19.0 kcal/mol for non-minimized and minimized complexes, respectively. Lastly, comparing the results obtained with the Charmm27 and OPLS force fields, we observed that the mean of OPLS is less positive as compared to the mean of the energy distribution calculated with Charmm27. Thus, these results suggest that the OPLS force field resulted in less positive ΔΔG_el _(less unfavorable energies), and the Charmm27 and Amber98 force fields resulted in the most unfavorable ΔΔG_el _in the hetero-complex cases.

In addition to the above observations, in some exceptional cases the ΔΔG_el _calculated with different force fields differed by more than 100 kcal/mol. Particular example is tail-associated lysozyme bound to baseplate structural protein GP27 (PDB ID 1k28) for which ΔΔG_el _calculated with Charmm27, e(in) = 1.0 and probe radius 1.4A was 261.7 kcal/mol while it was calculated to be 391.1 kcal/mol with Amber98 force field. We have not investigated these exceptions in detail; however, the differences reported in Tables 3, 4 as "Min" and "Max" indicate such cases. In many cases, even a difference of several kcal/mol could be crucial for getting the correct biophysics of the binding.

## Discussion

We have done extensive testing of the sensitivity of the electrostatic component of the binding free energy with respect to internal dielectric constant values, probe radius, and several commonly used force fields. The goal was also to reveal the general trends and to elaborate on the role of electrostatics on the binding for hetero- and homo-complexes. The study was done on a very large set of protein-protein complexes: 260 hetero-complexes and 2148 homo-complexes. Energy minimization and full-scale electrostatic calculations have never been done on such a large dataset before. This ensures that the obtained results and conclusions are statistically significant.

A common practice is to validate the results of numerical simulations against experimental data. However, it is impossible to experimentally determine the electrostatic component of the binding free energy. The binding free energy is made of a variety of energy terms whose interplay results in the observed affinity. Even the pH-dependence of the binding is not a pure electrostatic event[71]. The changes of the protonation states of ionizable groups induced by either pH changes or the binding in turn cause conformational changes and thus alter the contribution of non-electrostatic energy terms to the binding affinity as demonstrated in a recent study[71]. The most successful attempt so far to compare electrostatic calculations to experimental data was done by Dong and Zhou[47]. They carried out Poisson-Boltzmann calculations on a set of 64 mutations over six protein-protein complexes and compared their predictions with experimental data. The mutants were selected to involve charge-charge interactions across the interface of the corresponding complex and thus suggested the putative dominance of electrostatic interactions over the rest of the energy terms contributing to the binding. It was shown that the calculations done with a zero probe radius and an internal dielectric constant of 4.0 provided the closest agreement with experimental data. However, as pointed out by the authors, other contributions, such as van der Waals interactions and hydrophobic effect, may play important role[47]. Modeling the non-electrostatic contributions on such a large set of data as ours could introduce significant noise and obscure the results. That is our reasoning for not attempting to compare our calculations to cases in which the total binding free energy was experimentally measured. Proper modeling of the absolute binding free energy requires that the entropy be also accounted for, a task that is not easily achieved, especially on a large set of data.

Despite the fact that electrostatics is only one of the many forces contributing to the binding, in many cases, its involvement could be related to biologically important effects. For this reason, many researchers employ electrostatic calculations to find out the electrostatic component of the binding free energy. However, the value of the dielectric constant, the method of determining the molecular surface (probe radius), and the force field parameters are still a personal preference. Our study demonstrated that the absolute value of the calculated ΔΔG_el _is very sensitive to all of the above-mentioned parameters. While this is expected to be the case for different internal dielectric constants and to some extent for different probe radii, the observation that the results are quite sensitive to the force field parameters deserves attention. The average difference of the ΔΔG_el _calculated with different force fields can be as large as 20 kcal/mol and more. It seems to us that the energy minimization with the corresponding force field does not make much difference. Since the minimization protocol minimizes the total energy and not just the electrostatic component, the outcome for electrostatic energy will vary case by case. All of these observations indicate that the absolute values of the ΔΔG_el _should be considered with precaution.

This study revealed a major difference between hetero- and homo-complexes with respect to the calculated ΔΔG_el_. The calculated electrostatic component of the binding free energy opposes binding for approximately 80% of the homo-complex cases, despite the internal dielectric constant, probe radius, and force field. In contrast, the role of electrostatics on the binding of hetero-complexes depends on all of the factors above. Using a low internal dielectric constant and a probe radius of 1.4A, despite the force field used, most of the calculated ΔΔG_el _opposes binding. However, increasing the internal dielectric constant to 20 or decreasing the probe radius to R = 0.0A results in 60% of the cases having a ΔΔG_el _favoring the binding. These findings offer a pragmatic prescription for assessing the confidence of the predicted role of ΔΔG_el _on the binding affinity. It seems that despite the differences between the homo- and hetero-complexes, if the binding free energy is calculated to be a large positive quantity, then the conclusions could be considered independent from the choice of the parameters. However, cases in which the ΔΔG_el _are calculated with a particular set of parameters and corresponding magnitude is a small number close to zero are more complicated. Our study indicates that the calculated ΔΔG_el _could easily change sign upon changing the parameters of the protocol or upon switching the force field. Therefore, the conclusions made for the role of electrostatics on the binding in case of a small ΔΔG_el _should be carefully investigated by varying the force fields and the parameters of the computational protocol.

Our study, being applied on 2408 protein-protein complexes, offers the possibility to statistically address the question of what role electrostatics play in protein-protein binding. A similar question of what role salt bridges play on protein stability was extensively studied by Tidor and co-workers [78-80]. It was concluded that in most cases, salt bridges destabilize proteins. They also investigated the effect of electrostatic interactions on protein binding since salt bridges are also formed across protein-protein interfaces[81]. It was found that charge interactions in barnase-barstar complexes are optimized to favor the binding[25,26]. Schreiber and co-workers[22,82,83] employed experimental and computational methods to investigate the role of electrostatic interactions on barnase-barstar association. It was shown that charge-charge interactions can be re-designed to result in tighter binding[30]. The association rate was also studied on a set 68 transient hetero-complexes, demonstrating that electrostatics plays a marginal role[84]. Recently the association rates of four protein complexes and twenty three mutants were modeled by Zhou and co-workers [85]. It was shown that the calculations with vdW surface presentation (zero probe radius) and non-linear PB equation can successfully reproduce experimental data, while the models with molecular surface presentation (probe radius 1.4A) gave nonphysical results. The role of electrostatic interactions on the stability of monomeric protein was also systematically investigated. Makhatadze and co-workers have shown that so called "complex" salt bridges, in which the anchor residue forms hydrogen bonds with two or more opposite charged residues simultaneously, contribute significantly to protein stability [86]. This observation is valid not only for buried but for surface exposed groups as well [87-90]. It terms of different types of hydrophilic group, Pace and co-workers demonstrated that Asp and Glu amino acids contribute the most to protein stability [91], however the individual contributions are strongly context-dependent [92]. These observations and the successes in re-engineering of more stable proteins and tighter complexes than the wild type, demonstrate that the current computational methods can adequately model electrostatic interactions in biological macromolecules. However, it should be pointed out that the effect of re-designing is calculated and measured in respect to a reference state, the wild type protein or protein complex. Thus, an increased stability or tighter binding due to electrostatic optimization does not necessary mean that electrostatics plays favorable role. The only conclusion that can be made is that the re-engineering makes electrostatic contribution either more favorable or less unfavorable.

The results of our study indicate that the role electrostatics have on protein-protein binding in hetero-complexes cases is the same as the role salt bridges have on protein stability; i.e., in some cases, it will favor the binding, but in other cases it will oppose the binding. In our previous study [93] we have shown that electrostatic component of the binding free energy tends to be optimized in respect to random shuffling of the amino acid sequence of the corresponding partners. It was also demonstrated that the salt dependence of the binding is not correlated with macroscopic parameters of the monomers [63,94]. Taking together these observations, the findings of above mentioned studies and the results presented in this work, it could be stated that in majority of the cases electrostatics favor hetero-complexes formation. However, the role of electrostatics for each individual case depends on the fine details at the interface as formation of "complex" salt bridges, for example. The situation is quite different for homo-complexes. As was shown, all of the computational protocols predicted that, in majority of the cases, the electrostatics opposes binding. Since it was stated that macroscopic parameters do not matter much for protein-protein binding, this is a surprising observation. However, Table 1 provides an explanation. The homo-complexes differ from hetero-complexes not only by their macroscopic parameters as being made of two monomer carrying the same charge, but their interfaces are also different. Only 8% of homo-complexes have opposite charged interfaces, while this percentage is 58% for hetero-complexes. In remaining 92% of the cases, homo-complexes are formed between interfaces carrying either the same net charge or not having net charge at all. Perhaps, formation of "complex" salt bridges [86] and favorable polar interaction is much more difficult in this case as compared with hetero-complexes.

In this work we have not considered possible protonation changes induced by the binding. It was done in order to reduce computational demand of the numerical protocol. However, protonation changes may be common phenomena in protein binding as demonstrated recently by Jensen and co-workers [95]. Our recent investigation [96] also indicates that from statistical point of view there is 70% chance that binding will cause proton uptake/release of more than 0.5 proton units at pH = 7.0. Adjusting the ionization states according to the predicted pKa's of ionizable groups will definitely make the calculated ΔΔG_el _more favorable (or less unfavorable) compared with calculations done with default charge states. However, most of the predicted ionization changes are fractional numbers and modeling of non-integer charge changes requires ensemble approach in computing ΔΔG_el_, a task which is quite difficult to accomplish on a set of 2408 protein complexes.

The study revealed that for a significant fraction of hetero-complexes and in vast majority of homo-complexes the electrostatics opposes binding. Since most of the protein-protein complexes in the cell are homo-complexes, the overall role of electrostatics is predicted to oppose binding. Instead, electrostatics provides the necessary specificity and steering, processes equally important for protein-protein association.

## Conclusion

The analysis of the sensitivity of the ΔΔG_el_, calculated with different force fields, internal dielectric constants, probe radius value and minimization protocols, gives us the opportunity to suggest a set of rules of calculating (a) the absolute value of ΔΔG_el _and (b) the sign of ΔΔG_el_: (a1) if there is no prior knowledge what the effective value of both internal dielectric constant and probe radius are for a given protein complex and a particular protocol of calculating the ΔΔG_el_, then the absolute value of calculated ΔΔG_el _is meaningless; (a2) provided that the effective value of both internal dielectric constant and probe radius are known for a given protein complex and a particular protocol of calculating the ΔΔG_el_, then the absolute value of ΔΔG_el _should be obtained as an average of ΔΔG_el _calculated with different force fields; (a3) energy minimization does not help in obtaining consistent ΔΔG_el_'s, since it minimizes the total energy, not just the electrostatic component. Therefore, the absolute value of ΔΔG_el _should be obtained as an average of ΔΔG_el _calculated with different force fields, provided that other parameters are known; (b1) if there is no prior knowledge what the effective value of both internal dielectric constant and probe radius are for a given protein complex and a particular protocol of calculating the ΔΔG_el_, then the sign of ΔΔG_el _(determining the role of electrostatics on binding) is meaningful only if the absolute ΔΔG_el _calculated with either high internal dielectric constant or zero probe radius is larger than 1 kcal/mol. In case of hetero-complexes, if ΔΔG_el _is calculated to be positive and larger than 1 kcal/mol with ε(in) = 20 and probe radius 1.4A with given force field, then the probability of remaining positive in the calculations performed with different force fields is 0.99 (or 0.97 with ε(in) = 2). In case of homo-complexes, this probability is 0.95 (or 0.94 with ε(in) = 2); (b2) if there is a prior knowledge of the effective value of internal dielectric constant and probe radius, then calculations with a particular force field with and without minimization provide a good estimate of ΔΔG_el _sign in about 90% of the cases. In case of hetero-complexes, if ΔΔG_el _is calculated to be positive and larger than 1 kcal/mol with ε(in) = 20 and probe radius 1.4A with given force field, then the probability of remaining positive in the calculations performed with different dielectric constant is 0.98. Probability of change of the sign ΔΔG_el _using different probe radii is about 0.1. In case of homo-complexes, these probabilities are 0.96 and 0.87.

## Supplementary Material

Additional file 1 Correlation plots of the electrostatic component of the binding free energy. The plots show the correlations of the electrostatic component of the binding free energy calculated with different force fields. Click here for file

## References

[B1] Alberts B, Bray D, Lewis J, Raff M, Roberts K, Watson J (1994). Molecular Biology of the Cell.

[B2] Alexov E (2008). Protein-protein interactions. Curr Pharm Biotechnol.

[B3] Sham Y, Chu Z, Tao H, Warshel A (2000). Examining Methods for Calculations of Binding Free Energies: LRA, LIE, PDLD-LRA, and PDLD/S-LRA Calculations of Ligands Binding to an HIV Protease. Proteins.

[B4] Keskin O, Ma BY, Rogale K, Gunasekaran K, Nussinov R (2005). Protein-protein interactions: organization, cooperativity and mapping in a bottom-up Systems Biology approach. Phys Biol.

[B5] McDonald IK, Thornton JM (1994). Satisfying hydrogen bonding potential in proteins. J Mol Biol.

[B6] Jones S, Thornton J (1996). Principles of protein-protein interactions derived from structural studies. Proceedings of the National Academy of Sciences.

[B7] Sheinerman F, Norel R, Honig B (2000). Electrostatics Aspects of Protein-Protein Interactions. Current Opinion in Structural Biology.

[B8] Kundrotas PJ, Alexov E (2006). Electrostatic properties of protein-protein complexes. Biophys J.

[B9] Lensink MF, Mendez R (2008). Recognition-induced conformational changes in protein-protein docking. Curr Pharm Biotechnol.

[B10] Keskin O, Gursoy A, Ma B, Nussinov R (2008). Principles of Protein-Protein Interactions: What are the Preferred Ways For Proteins To Interact?. Chem Rev.

[B11] Jensen JH (2008). Calculating pH and salt dependence of protein-protein binding. Curr Pharm Biotechnol.

[B12] Bordner AJ, Abagyan R (2005). Statistical analysis and prediction of protein-protein interfaces. Proteins.

[B13] Jones S, Thornton J (1997). Prediction of Protein-Protein Interaction Sites using Patch Analysis. J Mol Biol.

[B14] Jones S, Thornton J (1996). Principles of protein-protein interactions. PNAS (USA).

[B15] Nooren IMA, Thornton JM (2003). Structural characterisation and functional significance of transient protein-protein interactions. Journal of Molecular Biology.

[B16] Albeck S, Schreiber G (1999). Biophysical characterization of the interaction of the beta-lactamase TEM-1 with its protein inhibitor BLIP. Biochemistry.

[B17] Dong F, Vijayakumar M, Zhou HX (2003). Comparison of calculation and experiment implicates significant electrostatic contributions to the binding stability of barnase and barstar. Biophys J.

[B18] Elcock A, Sept D, McCammon J (2000). Computer simulation of protein-protein interactions. J Phys Chem.

[B19] Elcock AH, Sept D, McCammon JA (2001). Computer simulation of protein-protein interactions. J Phys Chem B.

[B20] Clackson T, Wells JA (1995). A Hot-Spot of Binding-Energy in a Hormone-Receptor Interface. Science.

[B21] Wells JA (1991). Systematic Mutational Analyses of Protein Protein Interfaces. Method Enzymol.

[B22] Schreiber G, Fersht AR (1993). Interaction of barnase with its popypeptide inhibitor barstar studied by protein engineering. Biochemistry.

[B23] Schreiber G, Frisch C, Fersht AR (1997). The role of Glu73 of barnase in catalysis and the binding of barstar. J Mol Biol.

[B24] Massova I, Kollman PA (1999). Computational alanine scanning to probe protein-protein interactions: A novel approach to evaluate binding free energies. J Am Chem Soc.

[B25] Lee LP, Tidor B (2001). Optimization of binding electrostatics: charge complementarity in the barnase-barstar protein complex. Protein Sci.

[B26] Lee LP, Tidor B (2001). Barstar is electrostatically optimized for tight binding to barnase. Nat Struct Biol.

[B27] Schreiber G, Ferst A (1993). Interaction of Barnase with Its Polypeptide Inhibitor Barstar Studied by Protein Engineering. Biochemistry.

[B28] Frisch C, Schreiber G, Johnson CM, Fersht AR (1997). Thermodynamics of the interactions of barnase and barstar: changes in free energy *versus *changes in enthalpy on mutation. J Mol Biol.

[B29] Lee L, Tidor B (2001). Optimization of binding electrostatics: Charge complementarity in the barnase-barstar protein complex. Protein Sci.

[B30] Seizer T, Albeck S, Schreiber G (2000). Rational design of faster associating and tigher binding protein complexes. Nature Struc Biol.

[B31] Ma CS, Baker NA, Joseph S, McCammon JA (2002). Binding of aminoglycoside antibiotics to the small ribosomal subunit: A continuum electrostatics investigation. J Am Chem Soc.

[B32] Sims PA, Wong CF, McCammon JA (2004). Charge optimization of the interface between protein kinases and their ligands. J Comput Chem.

[B33] Sims PA, Wong CF, Vuga D, McCammon JA, Sefton BM (2005). Relative contributions of desolvation, inter- and intramolecular interactions to binding affinity in protein kinase systems. J Comput Chem.

[B34] Norel R, Sheinerman F, Petrey D, Honig B (2001). Electrostatic contribution to protein-protein interactions: Fast energetic filters for docking and their physical basis. Prot Sci.

[B35] Sheinerman FB, Honig B (2002). On the role of electrostatic interactions in the design of protein-protein interfaces. Journal of Molecular Biology.

[B36] Muegge I, Schweins T, Warshel A (1998). Electrostatic Contribution to Protein-Protein Binding Affinities: Application to Rap/Raf Interaction. Proteins.

[B37] Gohlke H, Kiel C, Case D (2003). Insights into protein-protein binding by binding free energy calculation and free energy decomposition for the Ras-Raf and Ras-RalGDS complexes. J Mol Biol.

[B38] Sharp KA (1994). Electrostatic Interactions in Macromolecules. Curr Opin Struct Biol.

[B39] Gilson M, Honig B (1986). The Dielectric Constant of a Folded Protein. Biopolymers.

[B40] Simonson T, Brooks C (1996). Charge Screening and the Dielectric Constant of Proteins: Insights from Molecular Dynamics. J Am Chem Soc.

[B41] Schulz C, Warshel A (2001). What Are the Dielectric "Constants" of Proteins and How To Validate Electrostatic Models. Proteins.

[B42] Brooks BR, Bruccoleri RE, Olafson BD, States DJ, Swaminathan S, Karplus M (1983). CHARMM: A program for macromolecular energy, minimization and dynamic calculations. J Comp Chem.

[B43] Gilson MK, Honig B (1991). The inclusion of electrostatic hydration energies in molecular mechanics calculations. J Comput Aided Mol Des.

[B44] Schiffer CA, Caldwell J, Stroud R, Kollman P (1992). Inclusion of Solvation Free Energy with Molecular Mechanics Energy: Alanine Dipeptide as a Test Case. Prot Sci.

[B45] Georgescu R, Alexov E, Gunner M (2002). Combining Conformational Flexibility and Continuum Electrostatics for Calculating Residue pKa's in Proteins. Biophys J.

[B46] Antosiewicz J, McCammon J, Gilson M (1994). Prediction of pH dependent properties of proteins. JMol Bio.

[B47] Dong F, Zhou H-X (2006). Electrostatic contribution to the binding stability of protein-protein complexes. Proteins.

[B48] Qin S, Zhou HX (2007). Do electrostatic interactions destabilize protein-nucleic acid binding?. Biopolymers.

[B49] Antosiewicz J, Briggs J, Elcock A, Gilson M, McCammon J (1996). Computing the ionization states of proteins with a detail charge model. J Comp Chem.

[B50] Nielsen J, Vriend G (2001). Optimizing the Hydrogen-Bond Network in Poisson-Boltzmann Equation-Based pKa Calculations. Proteins.

[B51] Nielsen J, Andersen K, Honig B, Hooft R, Klebe G, Vriend G, Wade R (1999). Improving macromolecular electrostatic calculations. Protein Eng.

[B52] Forrest L, Honig B (2005). An assessment of the accuracy of methods for predicting hydrogen positions in protein structures. Proteins.

[B53] Nielsen J, McCammon A (2003). On the evaluation and optimization of protein X-ray structures for pKa calculations. Protein Science.

[B54] Kundrotas PJ, Alexov E (2007). PROTCOM: searchable database of protein complexes enhanced with domain-domain structures. Nucleic Acids Res.

[B55] Li W, Jaroszewski L, Godzik A (2001). Clustering of highly homologous sequences to reduce the size of large protein databases. Bioinformatics.

[B56] Rocchia W, Sridharan S, Nicholls A, Alexov E, Chiabrera A, Honig B (2002). Rapid grid-based construction of the molecular surface and the use of induced surface charge to calculate reaction field energies: applications to the molecular systems and geometric objects. J Comput Chem.

[B57] Ponder JW (1999). TINKER-software tools for molecular design.

[B58] Brooks BR, Bruccoleri RE, Olafson BD, States DJ, Swaminathan S, Karplus M (1983). CHARMM: a program for macromolecular energy, minimization, and dynamics calculations. J Comput Chem.

[B59] Wang J, Wolf RM, Caldwell JW, Kollman PA, Case DA (2004). Development and testing of a general amber force field. J Comput Chem.

[B60] Jorgensen WL, Tirado-Rives J (1988). The OPLS potential function for proteins. J Am Chem Soc.

[B61] Still WC, Tempczyk A, Hawley RC, Hendrickson T (1990). Semianalytical Treatment of Solvation for Molecular Mechanics and Dynamics. J Am Chem Soc.

[B62] MacKerell AD, Bashford D, Bellot M, Dunbrack RL, Evanseck JD, Field MJ, Fischer S, Gao J, Guo H, Ha S (1998). All-atom empirical potential for molecular modeling and dynamics studies of proteins. J Phys Chem.

[B63] Bertonati C, Honig B, Alexov E (2007). Poisson-Boltzmann calculations of nonspecific salt effects on protein-protein binding free energies. Biophys J.

[B64] Rocchia W, Alexov E, Honig B (2001). Extending the applicability of the nonlinear Poisson-Boltzmann equation: Multiple dielectric constants and multivalent ions. J Phys Chem.

[B65] Rocchia W, Sridharan S, Nicholls A, Alexov E, Chiabrera A, Honig B (2002). Rapid Grid-based Construction of the Molecular Surface and the Use of Induced Surface Charges to Calculate Reaction Field Energies: Applications to the Molecular Systems and Geometrical Objects. J Comp Chem.

[B66] Nielsen JE, McCammon JA (2003). Calculating pKa values in enzyme active sites. Protein Science.

[B67] Gomez J, Freire E (1995). Thermodynamic Mapping of the Inhibitor Site of the Aspartic Protease Endothiapepsin. J Mol Biol.

[B68] Elcock A, McCammon A (1998). Electrostatic Contributions to the Stability of Halophilic Proteins. J Mol Biol.

[B69] Sharp KA (1996). Electrostatic interactions in hirudin-thrombin binding. Biophys Chem.

[B70] Trylska J, Antosiewich J, Geller M, Hodge C, Klabe R, Head M, Gilson M (1999). Thermodynamic linkage between the binding of protons and inhibitors to HIV-1 protease. Protein Science.

[B71] Alexov E (2004). Calculating Proton Uptake/Release and the Binding Free Energy Taking into Account Ionization and Conformation Changes Induced by Protein-Inhibitor Association. Application to Plasmepsin, Cathepsin D and Endothiapepsin-Pepstatin Complexes. Proteins.

[B72] Richards FM (1977). Areas, volumes, packing and protein structure. Ann Rev Biophys Bioeng.

[B73] Vijayakumar M, Zhou H (2001). Salt Bridges Stabilize the Folded State of Barnase. J Phys Chem.

[B74] Sham Y, Chu Z, Warshel A (1997). Consistent Calculations of pKa's of Ionizable Residues in Proteins: Semi-microscopic amd Microscopic Approaches. J Phys Chem.

[B75] Roca M, Messer B, Warshel A (2007). Electrostatic contributions to protein stability and folding energy. FEBS Lett.

[B76] Warshel A, Sharma PK, Kato M, Parson WW (2006). Modeling electrostatic effects in proteins. Biochim Biophys Acta.

[B77] Alexov E (2003). The role of the protein side chain fluctuations on the strength of pair wise electrostatic interactions. Comparing experimental with computed pKa's. Proteins.

[B78] Hendsch Z, Tidor B (1994). Do salt bridges stabilize proteins? A continuum electrostatics analysis. Prot Sci.

[B79] Luisi DL, Snow CD, Lin JJ, Hendsch ZS, Tidor B, Raleigh DP (2003). Surface salt bridges, double-mutant cycles, and protein stability: an experimental and computational analysis of the interaction of the Asp 23 side chain with the N-terminus of the N-terminal domain of the ribosomal protein l9. Biochemistry.

[B80] Hendsch ZS, Sindelar CV, Tidor B (1998). Parameter dependence in continuum electrostatic calculations: a study using protein salt bridges. J Phys Chem.

[B81] Kangas E, Tidor B (1999). Charge optimization leads to favorable electrostatic binding free energy. Phys Rev E Stat Phys Plasmas Fluids Relat Interdiscip Topics.

[B82] Martinez JC, Filimonov VV, Mateo PL, Schreiber G, Fersht AR (1995). A calorimetric study of the thermal-stability of barstar and its interaction with barnase. Biochemistry.

[B83] Schreiber G, Fersht AR (1995). Energetics of protein-protein interactions: analysis of the barnase-barstar interface by single mutations and double mutant cycles. J Mol Biol.

[B84] Shaul Y, Schreiber G (2005). Exploring the charge space of protein-protein association: A proteomic study. Proteins-Structure Function and Bioinformatics.

[B85] Alsallaq R, Zhou HX (2008). Electrostatic rate enhancement and transient complex of protein-protein association. Proteins.

[B86] Gvritishvili AG, Gribenko AV, Makhatadze GI (2008). Cooperativity of complex salt bridges. Protein Sci.

[B87] Schweiker KL, Zarrine-Afsar A, Davidson AR, Makhatadze GI (2007). Computational design of the Fyn SH3 domain with increased stability through optimization of surface charge charge interactions. Protein Sci.

[B88] Gribenko AV, Makhatadze GI (2007). Role of the charge-charge interactions in defining stability and halophilicity of the CspB proteins. J Mol Biol.

[B89] Strickler SS, Gribenko AV, Gribenko AV, Keiffer TR, Tomlinson J, Reihle T, Loladze VV, Makhatadze GI (2006). Protein stability and surface electrostatics: a charged relationship. Biochemistry.

[B90] Makhatadze GI, Loladze VV, Ermolenko DN, Chen X, Thomas ST (2003). Contribution of surface salt bridges to protein stability: guidelines for protein engineering. J Mol Biol.

[B91] Trevino SR, Scholtz JM, Pace CN (2007). Amino acid contribution to protein solubility: Asp, Glu, and Ser contribute more favorably than the other hydrophilic amino acids in RNase Sa. J Mol Biol.

[B92] Takano K, Scholtz JM, Sacchettini JC, Pace CN (2003). The contribution of polar group burial to protein stability is strongly context-dependent. J Biol Chem.

[B93] Brock K, Talley K, Coley K, Kundrotas P, Alexov E (2007). Optimization of electrostatic interactions in protein-protein complexes. Biophys J.

[B94] Talley K, Alexov E (2008). Modelling Salt Dependence of Protein-Protein Association:Linear vs Non-Linear Poisson-Bolzmann Equation. Comm in Computational Physics.

[B95] Mason AC, Jensen JH (2007). Protein-protein binding is often associated with changes in protonation state. Proteins.

[B96] Mitra R, Shyam R, Mitra I, Miteva MA, Alexov E (2008). Calculation of the proponation states of proteins and small molecules: Inmplications to ligand-receptor interactions. Current computer-aided drug design.

