# Supplementary material for: On the electrostatic component of protein-protein binding free energy
Source: PMC Biophys. 2008 Nov 5;1:2. doi: 10.1186/1757-5036-1-2 (PMC2666630; doi:10.1186/1757-5036-1-2)
Supplement: Additional file 1 — Correlation plots of the electrostatic component of the binding free energy. The plots show the correlations of the electrostatic component of the binding free energy calculated with different force fields. [file 1757-5036-1-2-S1.doc]

**Additional file 1**

This section provides the correlation plots of ΔΔG_el_ calculated with different force fields.

**Fig. 1S**. ΔΔG_el_ calculated with Charmm27 force field parameters versus ΔΔG_el_ calculated with Amber98. The internal dielectric constant is 2.0 and the molecule surface is determined with probe radius 1.4 A. Non-minimized hetero-complexes.

**Fig. 2S**. ΔΔG_el_ calculated with Charmm27 force field parameters versus ΔΔG_el_ calculated with Amber98. The internal dielectric constant is 2.0 and the molecule surface is determined with probe radius 0.0 A. Non-minimized hetero-complexes.

**Fig. 3S**. ΔΔG_el_ calculated with OPLS force field parameters versus ΔΔG_el_ calculated with Amber98. The internal dielectric constant is 2.0 and the molecule surface is determined with probe radius 1.4 A. Non-minimized hetero-complexes.

**Fig. 4S**. ΔΔG_el_ calculated with OPLS force field parameters versus ΔΔG_el_ calculated with Amber98. The internal dielectric constant is 2.0 and the molecule surface is determined with probe radius 0.0 A. Non-minimized hetero-complexes.

**Fig. 5S**. ΔΔG_el_ calculated with OPLS force field parameters versus ΔΔG_el_ calculated with Charmm27. The internal dielectric constant is 2.0 and the molecule surface is determined with probe radius 1.4 A. Non-minimized hetero-complexes.

**Fig. 6S**. ΔΔG_el_ calculated with OPLS force field parameters versus ΔΔG_el_ calculated with Charmm27. The internal dielectric constant is 2.0 and the molecule surface is determined with probe radius 0.0 A. Non-minimized hetero-complexes.

**Fig. 7S**. ΔΔG_el_ calculated with Charmm27 force field parameters versus ΔΔG_el_ calculated with Amber98. The internal dielectric constant is 2.0 and the molecule surface is determined with probe radius 1.4 A. Non-minimized homo-complexes.

**Fig. 8S**. ΔΔG_el_ calculated with Charmm27 force field parameters versus ΔΔG_el_ calculated with Amber98. The internal dielectric constant is 2.0 and the molecule surface is determined with probe radius 0.0 A. Non-minimized homo-complexes.

**Fig. 9S**. ΔΔG_el_ calculated with OPLS force field parameters versus ΔΔG_el_ calculated with Amber98. The internal dielectric constant is 2.0 and the molecule surface is determined with probe radius 1.4 A. Non-minimized homo-complexes.

**Fig. 10S.** ΔΔG_el_ calculated with OPLS force field parameters versus ΔΔG_el_ calculated with Amber98. The internal dielectric constant is 2.0 and the molecule surface is determined with probe radius 0.0 A. Non-minimized homo-complexes.

**Fig. 11S**. ΔΔG_el_ calculated with OPLS force field parameters versus ΔΔG_el_ calculated with Charmm27. The internal dielectric constant is 2.0 and the molecule surface is determined with probe radius 1.4 A. Non-minimized homo-complexes.

**Fig. 12S**. ΔΔG_el_ calculated with OPLS force field parameters versus ΔΔG_el_ calculated with Charmm27. The internal dielectric constant is 2.0 and the molecule surface is determined with probe radius 0.0 A. Non-minimized homo-complexes.
